# Evaluation of Phenolic Content Variability along with Antioxidant, Antimicrobial, and Cytotoxic Potential of Selected Traditional Medicinal Plants from India

**DOI:** 10.3389/fpls.2016.00407

**Published:** 2016-03-31

**Authors:** Garima Singh, Ajit K. Passsari, Vincent V. Leo, Vineet K. Mishra, Sarathbabu Subbarayan, Bhim P. Singh, Brijesh Kumar, Sunil Kumar, Vijai K. Gupta, Hauzel Lalhlenmawia, Senthil K. Nachimuthu

**Affiliations:** ^1^Department of Biotechnology, Mizoram UniversityAizawl, India; ^2^Sophisticated Analytical Instrument Facility, CSIR-Central Drug Research InstituteLucknow, India; ^3^Molecular Glyco-Biotechnology Group, Discipline of Biochemistry, National University of Ireland GalwayGalway, Ireland; ^4^Department of Pharmacy, Regional Institute of Paramedical and Nursing Sciences ZemabawkAizawl, India

**Keywords:** antioxidants, antimicrobial, HPLC-DAD-ESI-TOF-MS, phenolic compounds, medicinal plants, cytotoxicity

## Abstract

Plants have been used since ancient times as an important source of biologically active substances. The aim of the present study was to investigate the phytochemical constituents (flavonoids and phenolics), antioxidant potential, cytotoxicity against HepG2 (human hepato carcinoma) cancer cell lines, and the antimicrobial activity of the methanol extract of selected traditional medicinal plants collected from Mizoram, India. A number of phenolic compounds were detected using HPLC-DAD-ESI-TOF-MS, mainly Luteolin, Kaempferol, Myricetin, Gallic Acid, Quercetin and Rutin, some of which have been described for the first time in the selected plants. The total phenolic and flavonoid contents showed high variation ranging from 4.44 to 181.91 μg of Gallic Acid equivalent per milligram DW (GAE/mg DW) and 3.17 to 102.2 μg of Quercetin/mg, respectively. The antioxidant capacity was determined by DPPH (IC_50_ values ranges from 34.22 to 131.4 μg/mL), ABTS (IC_50_ values ranges from 24.08 to 513.4 μg/mL), and reducing power assays. Antimicrobial activity was assayed against gram positive (*Staphylococcus aureus*), gram negative (*Escherichia coli, Pseudomonas aeruginosa*), and yeast (*Candida albicans*) demonstrating that the methanol extracts of some plants were efficacious antimicrobial agents. Additionally, cytotoxicity was assessed on human hepato carcinoma (HepG2) cancer cell lines and found that the extracts of *Albizia lebbeck, Dillenia indica*, and *Bombax ceiba* significantly decreased the cell viability at low concentrations with IC_50_ values of 24.03, 25.09, and 29.66 μg/mL, respectively. This is the first report of detection of phenolic compounds along with antimicrobial, antioxidant and cytotoxic potential of selected medicinal plants from India, which indicates that these plants might be valuable source for human and animal health.

## Introduction

Use of synthetic food additives imposed the need for the search of a natural alternative to synthesize antioxidants. For this, medicinal plants are well known to be used as natural antioxidant agents, as they possess low toxicity and are rich sources of pharmaceutical compounds (Carocho and Ferreira, 2013). Epidemiological studies have shown that the plants abundant in active secondary metabolites with antioxidant and antimicrobial properties can be exploited for bioactive compounds. Natural antioxidants such as polyphenols, tocopherols, carotenoids, ascorbic acid etc. could prevent oxidation reactions which may help in restoring the quality of food products (Wojcik et al., 2010). The antioxidant activity of medicinal plants is generally studied with respect to total phenolic compounds and their free radical scavenging assays as they may be responsible for various bioactivities (Farhat et al., 2013; Navas-Lopez et al., 2014; Iqbal et al., 2015).

Emerging resistance against bacterial and fungal species results in a serious decrease in effective antimicrobials. Hence, industries are tending to reduce the use of chemical preservatives and to adopt natural preservatives (Nychas, 1995). Medicinal plants and their metabolites are the natural sources to be used as antimicrobials. Several researchers have reported the use of plant compounds against different types of bacterial pathogens, including food borne pathogens as well (Kukić et al., 2008). There is also a need of new, efficient anticancer drugs with reduced side effects and traditional medicinal plants have been proved a promising source for such entities (DeSantis et al., 2014). Among the potential molecules recovered from plants, over 60% of anti-cancer drugs directly or indirectly are originated from plants including paclitaxel (Taxol), paclitaxel, curcumin, and cannabinoids (Gordaliza, 2007). Plant-derived substances endowed with anticancer and chemoprevention activity is being recently reviewed by Fridlender et al. (2015).

Medicinal plants with ethnomedicinal history collected from North East India are being used as biomarkers in folklore medicine for the treatment of various diseases like diabetes, diarrhea, hypertension, cancer, etc. (Sharma et al., 2001; Mishra et al., 2013). From the collected plants, 12 best known plants (*Abroma augusta, Albizia chinensis, Albizia lebbeck, Bombax ceiba, Callicarpa arborea, Chonemorpha fragrans, Clerodendrum Colebrookianum, Costus speciosus, Dillenia indica, Gynura conyza, Hibiscus sabdariffa, Momordica charantia*) based on their ethnomedicinal knowledge, wide usage, and local availability were selected for their antioxidant, antimicrobial activities, cytotoxicity screening, and phenolic compounds determination (Table 1). Among the selected plants, *D. indica* leaf extract has shown to possess effective antidiabetic and antihyperlipidemic activities (Kumar et al., 2011a,b), antimicrobial and brime shrimp lethality (Apu et al., 2010). The *Albizia* species leaf extract was proven to be bioactive due to the presence of saponins, tannins, sterols, phenolics, and polysaccharides (Yanishlieva et al., 2006; Liu et al., 2009). The leaf extract of *A. augusta* has been shown to attenuate diabetes induced nephropathy as well as cardiomyopathy by inhibition of oxidative stress and inflammatory response (Khanra et al., 2015). The extracts exhibited significant antimicrobial activity against both gram positive and gram negative bacterial and fungal pathogens as well as cytotoxicity against brine shrimp nauplii (Saikot et al., 2012). Chemical constituents obtained from leaves of *M. charantia* showed hypoglycemic effect and can promote the release of insulin (Ng et al., 1986; Hui et al., 2009).

**Table 1:** **General characteristics and pharmacological activities of the selected medicinal plants**.

**Voucher Number**	**Plant name**	**Common name**	**Family**	**Traditional medicinal uses**	**Pharmacological activities reported**
MZU/BT/14	*Abroma augusta* (Linn.)	Devil's cotton	*Malvaceae*	Menstrual disorders, sterility, nervous disorders, diabetes, and gonorrhea	Antidiabetic (Islam et al., 2012; Khanra et al., 2015)
MZU/BT/15	*Albizia chinensis* (Osbeck)	Silk tree	*Fabaceae*	Skin diseases, cut, and inflammations	Cytotoxic (Liu et al., 2009)
MZU/BT/16	*Albizia lebbeck* (L.) Benth.	Lebbeck (Flea tree	*Fabaceae*	Ulcer treatment, cold, cough, and respiratory disorders	Anti-arthritis (Pathak et al., 2009); antiallergic (Venkatesh et al., 2010); antimicrobial (Rahul et al., 2010)
MZU/BT/17	*Bombax ceiba* (Linn.)	Cotton tree	*Malvaceae*	Leaf juice is used to treat diarrhea	Anticancer and Antioxidants (Tundis et al., 2014); Diuretic (Jalalpure and Gadge, 2011)
MZU/BT/18	*Callicarpa arborea* Roxb.	Beautyberry Tree	*Verbenaceae*	Bark juice is used on cuts and wounds, abdominal colic	–
MZU/BT/19	*Chonemorpha fragrans* (Moon) Alst.	Frangipani vine	*Apocynaceae*	Stomach disorders, fever	Antidiabetic (Shende et al., 2009)
MZU/BT/20	*Clerodendrum colebrookianum* Walp.	Glory Bower	*Lamiaceae*	Hypertension, diabetes, and abdominal disorders	Anti-inflammatory (Deb et al., 2013); antidiabetic (Devi and Sharma, 2004).
MZU/BT/21	*Costus speciosus* Koen ex. Retz	Crepe ginger	*Costaceae*	Diabetes, diarrhea, fever, jaundice, and inflammations	Anticancer (Nair et al., 2014; Selim and Al-Jaouni, 2015).
MZU/BT/22	*Dillenia indica* (L.)	Elephant apple	*Dillaniaceae*	Diarrhea, cancer	Antidiabetic (Kumar et al., 2011a,b)
MZU/BT/23	*Gynura conyza* Cass.		*Asteraceae*	Leaf juice is used for wounds, ulcers, dysentery, Jaundice	–
MZU/BT/24	*Hibiscus sabdariffa* (Linn.)	Roselle	*Malvaceae*	Hypertension, liver diseases, urinary disorders, and fever	Antioxidant, Anticancer (Worawattananutai et al., 2014; Chiu et al., 2015)
MZU/BT/25	*Momordica charantia*	Bitter gourd	*Cucurbitaceae*	Digestive disorders, cholera, jaundice, diabetes, hypertension	Antidiabetic (Manik et al., 2013; Wang et al., 2014; Xu et al., 2015); Hypoglycemic (Nkambo et al., 2013); Antimicrobial (Yaldiz et al., 2015); Anticancer (Weng et al., 2013)

Although, most of the plants included in our study have been investigated elsewhere for their chemical constituents and/or for their antioxidant capability, to the best of our knowledge this is the first study focusing on the correlation thereof. In addition, our hypothesis to screen the antimicrobial and cytotoxicity is that it is appropriate to analyze samples from Mizoram, North East India which falls under Indo-Burma biodiversity hot spot region (Myers et al., 2000), as the environmental conditions can be effective on chemical composition of the plants with respect to antioxidant contents (Simirgiotis, 2013).

The aim of the present work is to detect the major phenolic compounds by HPLC-DAD- ESI-TOF-MS of the methanolic leaf extracts of selected 12 medicinal plants and to determine the total phenolics and flavanoids contents, antioxidant potential, ability to inhibit human hepato carcinoma (HepG2) cancer cell lines as well as their antimicrobial activity against selected bacterial and yeast pathogens.

## Materials and methods

### Reagents and chemicals

All chemicals used in the present study were of analytical HPLC grade and were purchased from Hi-media, India and Sigma-Aldrich, USA. ABTS (2,2- Azinobis-3-ethylbenzothiazoline-6-sulfonic acid), 2,2-diphenyl-1-picrylhydrazyl (DPPH), Dimethyl Sulfoxide, Sodium acetate trihydrate ACS, Ferric chloride hexahydrate A.R., Ferrous sulfate heptahydrate A.R., Folin ciocalteu's reagent L.R., Gallic acid monohydrate, L-Ascorbic acid A.R., Acetic acid glacial A.R., Sodium carbonate ACS, Potassium persulphate A.R. were purchased from Hi-media (India). 6-hydroxy-2,5,7,8-tetramethylchromane-2-carboxylic acid (trolox), Aluminum chloride AR, and Quercetin ≥ 95% (HPLC) solid were purchased from Sigma-Aldrich (USA). All chemicals and reagents were used without any further purification.

### Plant material and extraction

Fresh and healthy leaves of selected plants were collected from the Dampa Tiger Reserve forest [Dampa TRF] (23°25′N; 92°20′E), Mizoram, Northeast India during November 2014. Permission for the collection of medicinal plants from Dampa TRF was obtained from The Chief wildlife warden, Environment and forest department, Government of Mizoram, India. The selection of plant species was based on their ethanobotanical history and abundance (Table 1). Plants were identified at Department of Forestry, Mizoram University and voucher specimens were kept under the reference numbers (Table 1). Collected leaves were dried at room temperature in a well ventilated room and ground to fine powder in a domestic mixer grinder. The powder obtained was weighed and extracted in methanol for 48 h. The extract was filtered through a Whatmann no. 1 filter paper thrice and filtrate was evaporated to dryness at 40°C under reduced pressure by using a rotary evaporator (BUCHI, Switzerland) to obtain the crude extract. The extracts were kept at 4°C until further use.

### Phytochemical analysis

#### Determination of total phenolic content (TPC)

The content of total phenolic compounds of crude methanol extracts was determined spectrophotometrically by using Folin- ciocalteu assay (Attard, 2013). An aliquot of 30 μl extracts with varying concentration (10–100 mg/mL) was mixed with 150 μl of freshly prepared Folin reagent (1:10 v/v in water) and allowed to stand at 25°C for 5 min, and thereafter 120 μl sodium bicarbonate (75 gm/L) solution was added to the mixture. The mixture was allowed to incubate at 25°C for 90 min and absorbance was measured at 725 nm using a microplate spectrophotometer UV-vis (Multiscan™ GO, Thermo Scientific, MA, USA). Gallic acid (0–500 mg/L) was used to prepare the standard curve, which showed the linear regression of *r*^2^ > 0.99, and the level of phenolics was expressed in term of Gallic Acid equivalent per gram of plant extract. The measurements were done in triplicate.

#### Determination of total flavonoids content

The total flavonoids content was determined by using a colorimetric method (Chang et al., 2002). The standard curve of Quercetin solution in methanol was prepared with concentrations ranging from 0 to 500 μg/mL and absorbance was recorded at 420 nm with a microplate spectrophotometer UV-vis (Multiscan™ GO, Thermo Scientific, MA, USA). The flavonoid concentrations were expressed in μg quercetin equivalent per mg of extract.

### Determination of antioxidant activity

#### ABTS^+^ radical cation decoloration assay

ABTS^+^ radical scavenging capacity of the extract was measured with 96-well micotiter plate method (Re et al., 1999). Ascorbic acid was used as positive control, methanol as negative control and extract without ABTS as blank. The percentage of ABTS^+^ was calculated by using the formula:

(1) $$\begin{array}{ll} \begin{array}{l} \text{Scavenging capacity}\;(\%)\;=\;100-[(\text{A}-{\text{A}}_{\text{O}})\times 100/(\text{B}-{\text{B}}_{\text{O}})]; \end{array} & \left(1\right) \end{array}$$

Whereas, A = sample, A_O_ = sample blank, B = control, B_O_ = control blank.

IC_50_ values were calculated with the help of graph plotted as inhibition percentage against the concentration.

#### DPPH^+^ free radical scavenging assay

The ability of crude methanol extract to scavenge the DPPH free radical was determined by using the stable 2, 2-diphenyl-1-picrylhydrazyl radical (DPPH) (Re et al., 1999). An aliquot of 50 μl (of varying concentrations) was placed in 96-well microplate, and 200 μl of 0.1 mM DPPH dissolved in methanol was added and allowed to react at room temperature in the dark. The reduction of DPPH concentration was recorded by a decrease in absorbance at 515 nm till the absorbance stabilized (30 min). Ascorbic acid was used as positive control, methanol as negative control and extract without DPPH as blank. IC_50_ which represents the amount of antioxidant necessary to produce a 50% reduction of the DPPH was calculated with the calibration curve by linear regression. Results were expressed as a percentage reduction of DPPH absorption compared to control.

#### Reducing antioxidant power assay

Reducing power of the methanolic extracts was evaluated (Oyaizu, 1986). Extracts and ascorbic acid as standard of different concentration (10–1000 μg/mL) in 0.25 mL methanol were mixed with phosphate buffer (500 μl, 0.2 M, pH 6.6) and potassium ferricyanide [K_3_Fe (CN)_6_] (500 μl, 10 mg/mL). The mixture was mixed and incubated at 50°C for 20 min. After incubation, 500 μl of 10% trichloroacetic acid solution was added to each tube and the mixture was centrifuged at 8000 rpm for 10 min. Clear supernatant (100 μl) was mixed with equal amount of distilled water, 20 μl of ferric chloride (0.1% w/v) solution was added and absorbance was recorded at 700 nm. Reducing capacity of the extracts was linearly proportional to the concentration of a sample. Phosphate buffer was used as control.

### Antimicrobial assay

#### Test organisms

All plant extracts were screened against gram positive (*Staphylococcus aureus*: MTCC- 96), gram negative (*Escherichia coli:* MTCC- 739, *Pseudomonas aeruginosa:* MTCC-2453), and yeast (*Candida albicans:* MTCC-3017) by using agar well diffusion method (Rios et al., 1988). Test organisms were obtained from the Microbial Type Culture Collection (MTCC), Chandigarh, India and maintained on agar slants as per instructions. The bacterial inoculum was prepared to concentration of 1.0 × 10^4^ CFU/mL adjusted with sterile saline. The suspension was prepared fresh daily and stored at 4°C until use. The suspensions were spreaded on solid medium to verify the absence of contamination and to cross check the viability of inoculum. Three antibiotics ampicillin (10 mg/mL), streptomycin (10 mg/mL), and tetracycline (20 mg/mL) were used as positive control and solvents DMSO was used as negative control. Agar plates were incubated at 37°C for 24 h and the clear zone of inhibition in mm was taken as a degree of antimicrobial sensitivity. All experiments were done in triplicate and repeated thrice.

#### Determination of minimum inhibitory concentration (MIC) of plant extract

To determine the minimum inhibitory activity of extracts, the broth micro dilution technique using 96-well microtiter plate was used (Eloff, 1998). The bacterial suspension was adjusted to a final concentration of 1.0 × 10^−4^ CFU/mL (OD = 0.402). The plant extract was added at different concentrations (1–20 mg/mL) in 96-well microtiter plate containing a bacterial culture as test. Different concentration (1–20 mg/mL) of plant extract was used individually as specific controls. Four antibiotics along with bacterial culture were used as positive control. Solvent DMSO was used as negative control containing a bacterial culture. The plates were incubated at 37°C for 48 h and the absorbance was taken at 630 nm in spectrophotometer UV-vis (Multiscan™ GO, Thermo Scientific, MA, USA). IC_50_ was expressed as the concentration (mg/ml) of plant extract necessary to produce a 50% reduction of bacterial culture growth. It was calculated with the calibration curve by linear regression.

### Cytotoxicity activity

#### Cell lines and culture medium

Human hepato carcinoma (HepG2) cancer cell line was procured from National Centre for Cell Sciences (NCCS), Pune, India. Stock cells were cultured in DMEM supplemented with 10% inactivated Fetal Bovine Serum (FBS), penicillin (100 μg/mL), streptomycin (100 μg/mL), and amphotericin B (5 μg/mL) in a humidified atmosphere of 5% CO_2_ at 37°C until confluent. The cells were dissociated with a trypsin solution (0.2% trypsin, 0.02% EDTA, 0.05% glucose in PBS). The stock cultures were grown in 25 cm^2^ culture flasks and all experiments were carried out in 96 microtiter plates (Tarsons India Pvt. Ltd., Kolkata, India).

#### MTT assay

The cytotoxicity of the extracts was tested against human hepato carcinoma (HepG2) cell lines by the MTT reduction assay (Mosmann, 1983). HepG2 cell monolayer was trypsinized and seeded on 96-well microtiter plates with a cell density of approximately 10 × 10^−4^ cells per 100 μl of media in each well. The plates were incubated at 37°C for 24 h in 5% CO_2_ atmosphere. After incubation, the cells were treated with eight different concentrations (1, 10, 25, 50, 75, 100, 125, and 150 μg/mL) of crude methanol (0.5%) leaf extracts of *A. augusta, A. chinensis A. lebbeck, B. ceiba, C. arborea, C. colebrookianum, D. indica, G. conyza, C. speciosus, C. Fragrans, H. sabdariffa*, and *M. charantia*. Cells were incubated with 0.5% of methanol used as blank and untreated cells as a control was included for each sample. Each sample was performed in triplicate and cells were incubated for 72 h. After incubation, the culture medium was removed from each well by aspiration and 20 μl of MTT (3- [4, 5-dimethylthiazol-2-yl]-2, 5-diphenil-tetrazolium bromide, Sigma Chemical Co., USA, 5 mg/mL in PBS) was added to each well. After 4 h of incubation, DMSO (Himedia) was added to dissolve the purple formazan of MTT. The absorbance was measured by a microplate reader at a wavelength of 570 nm. The cell viability (%) was calculated using the formula-

(2) $$\begin{array}{l} \text{Cell viability }(\%)=(\text{OD sample – OD blank})\text{/}\\ \;(\text{OD control – OD blank})\times \text{100}\%, \end{array}$$

Where OD sample is the absorbance of the samples, OD blank is the absorbance of the blank (with the respective concentration solutions), and OD control is the absorbance of the control wells.

### Instrumentation

Analyses were carried out using an Agilent 1200 HPLC system interfaced with Agilent 6520 hybrid quadrupole time of flight mass spectrometer (Agilent Technologies, USA). 1200 HPLC system was equipped with quaternary pump (G1311A), online vacuum degasser (G1322A), Autosampler (G1329A), thermostatted column compartment (G1316C), and diode-array detector (G1315D).

#### Chromatographic conditions

Chromatographic separations were performed using a Thermo Betasil C_18_ column (250 × 4.5 mm, 5 μ) operated at 25°C employing a gradient elution using 0.3% formic acid in HPLC water (A) and acetonitrile (B) as mobile phase at a flow rate of 0.6 mL/min. The elusion consisted of a linear gradient from 20 to 50%; 0–10 min, 50–90%; 10–30 min, 90%; 30–35 then returned to the initial conditions over in 5 min. The sample injection volume was 10 μl.

#### Mass spectrometric condition

The mass spectrometer was operated in a positive electro spray ionization mode and spectra were recorded by scanning the mass range from *m/z* 50–1500 in both MS and MS/MS modes. Nitrogen was used as drying, nebulizing, and collision gas. The drying gas flow rate was 12 L/min. The heated capillary temperature was set to 350°C and nebulizer pressure at 45 psi. The source parameters capillary voltage (VCap), fragmentor, skimmer and octapole voltages were set to 3500, 175, 65, and 750 V, respectively. The accurate mass data of the molecular ions were processed through the Mass Hunter Workstation (version B 04.00) software.

### Statistical analysis

The relative viability of the treated cells compared to that of the control cells is expressed as percentage of cell viability. Statistical analysis was performed using Graph Pad (version 6.04; Graph Pad Software, Inc., La Jolla, CA, USA). The results were expressed in mean ± SD of the values obtained in triplicates from three independent experiments and analyzed by one way analysis of variance (ANOVA) followed by Duncan's multiple range tests for comparison of statistical significance (*P* < 0.05). Pearson correlation coefficients were calculated in order to measure the linear correlation between variables. All statistical calculations were performed by using SPSS software version 16.0. Regression analysis was used to determine inhibition concentration needed to inhibit 50% cell viability (IC_50_) by using thermo scientific Multiscan GO software.

## Results

### Determination of total phenolics (TPC), flavonoids (TFC), and antioxidant activity by DPPH, ABTS, and reducing power assays

The total phenol content (TPC) of all 12 plant extracts under study was found in ranging from 4.44 to 181.91 μg of GAE/mg as Gallic Acid equivalents. The maximum amount of TPC was recorded with the methanol extract of *B. ceiba* (181.91 μg of GAE/mg) followed by *D. indica* and *C. colebrookianum* with 180.15 and 158.37 μg of GAE/mg, respectively. Total flavonoids content (TFC) which was quantified as micro-gram of Quercetin equivalent per milligram of extracts ranges from 3.17 to 102.2 μg of Quercetin/mg. The highest TFC was found in *A. lebbeck* (102.2 μg of Quercetin/mg). The TFC of *D. indica* was 98.25 μg of Quercetin/mg and *C. fragrans* had the lowest TFC (3.17 μg of Quercetin/mg) among the 12 extracts estimated. This was followed by *C. speciosus* and *A. augusta* with TFC values of 6.61 and 9.22 μg of Quercetin/mg respectively.

**Figure 1:**
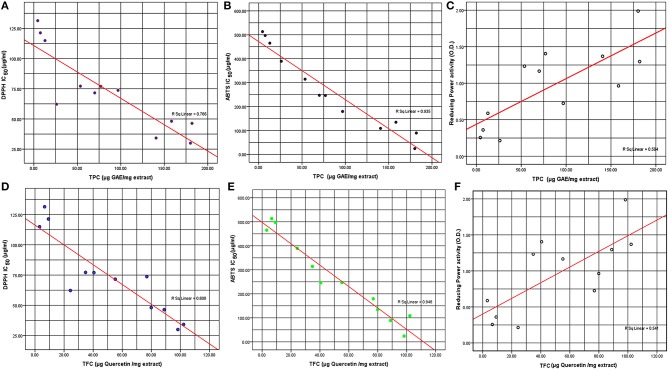
**Correlation of (A) TPC and DPPH free radical scavenging activity IC50 DPPH (μg, DPPH/mL), (B) TPC and ABTS (μg, ABTS/mL), (C) TPC and reducing power activity, (D) TFC and DPPH free radical scavenging activity IC50 DPPH (μg, DPPH/mL), (E) TFC and ABTS (μg, ABTS/mL), (F) TFC and reducing power activity**.

The antioxidant ability of the methanol extracts has been determined by using several assays (Table 2). IC_50_ values for DPPH and ABTS assays ranged from 34.22 to131.4 μg/mL and 24.08 to 513.4 μg/mL, respectively (Figure 1). The lower the IC_50_ value of plant extracts used, the higher was their free radical scavenging activity. Hence *D. indica* with an IC_50_ of 29.96 μg/mL in DPPH assay and 24.08 μg/mL of extract by ABTS assay indicates a significant antioxidant property in leaves of *D. indica*. Similarly, *A. lebbeck* also exhibited lower IC_50_ DPPH value of 34.22 μg/mL of extract; while a slightly higher value was observed for its ABTS IC_50_ which is 108.7 μg/mL of extract. This capability of *A. lebbeck* to be a natural antioxidant was substantiated by a reasonable ABTS and reducing power assay values. *B. ceiba* antioxidant potential of the methanol leaf extracts was also substantial with an IC_50_ DPPH value of 46.36 μg/mL of extract and ABTS IC_50_ value 89.19 μg/mL of extract. To the best of our knowledge, this is first reported antioxidant activity of ABTS in *A. augusta* (497.2 μg/mL), *C. fragrans* (465.2 μg/mL), *C. arborea* (314.4 μg/mL), *A. lebbeck* (108.7 μg/mL), *C. colebrookianum* (134.3 μg/mL), *B. ceiba* (89.19 μg/mL), *D. indica* (24.08 μg/mL), and *G. conyza* (246.3 μg/mL). Reducing power assay indicates the capacity of the compounds present in extracts antioxidant potentials. In the present study, the reducing power of all 12 extracts was verified for the concentration range (10–1000 μg/mL). At the highest concentration (1 mg/mL) tested, the increasing order of absorbance are as follows: 1.988 (*D. indica*) > 1.405 (*G. conyza*) > 1.369 (*A. lebbeck*) > 1.296 (*B. ceiba*) > 1.233 (*C. arborea*) > 0.9637 (*C. colebrookianum*) > 0.7244 (*H. sabdariffa*) (Figure 1). The rest of the remaining extracts under study showed negligible reducing power activity.

**Table 2:** **Correlation coefficient ***r***^**2**^ between antioxidant assays (DPPH, ABTS, and reducing power) and total phenolic content (TPC) and Total flavonoid content of selected medicinal plants**.

**Antioxidant assays**	**Corelation** ***r***^**2**^	
	**TPC**	**TFC**
DPPH	**0.766**	**0.808**
ABTS	**0.935**	**0.948**
Reducing power	0.594	0.541

*Bold values means significant correlations observed*.

From our result, significant correlation between the TPC and the free radical scavenging assays were obtained, which was proven by their correlation data with respect to TPC of *R*^2^ = 0.766, *R*^2^ = 0.935, and *R*^2^ = 0.594 with DPPH (IC_50_) values, ABTS (IC_50_) and reducing power (OD) values, respectively (Table 2). This indicates the influence of the electron donor's rich total phenols contents of the extracts under study, has impacted on their antioxidant capabilities. Since phenolic compounds present in the extracts are a good source of electron donors, they show reducing power. TPC and TFC produced a positive correlation as indicated by a *R*^2^ = 0.904; that clearly suggest that the flavonoids within the methanol extracts of the plants under study might be the major constituent in the total phenols obtained. In case of TFC also, significant correlation was found with ABTS (IC_50_) values of an *R*^2^ = 0.948; while a similar correlation was found with DPPH (IC_50_) values of *R*^2^ = 0.808 also (Table 2).

### Detection of phenolic compounds by HPLC-DAD-ESI-TOF-MS

The analysis of methanol extracts by using HPLC-DAD-ESI-TOF-MS showed the presence of a wide variety of polyphenols. Ions detected were tentatively identified by the molecular formula generated by the use of standard, if available, and after a thorough literature search. Table 3 listed the detected phenolic compounds with retention time, observed m/z, generated molecular formula and proposed compound detected in the methanol extracts of selected plants. The number of compounds detected as compared with the standard was in the range from 2 to 5 compounds in different plants under study along with the unidentified compounds detected (Table 3).

**Table 3:** **Detection of phenolic compounds by HPLC-DAD-ESI-TOF-MS in selected 12 plant extracts**.

**S. No**.	**RT (min)**	**Cal. [M+H]^+^**	**Obs. [M+H]^+^**	**Molecular formula**	**Error (ppm)**	**Compound name**	**Plant extract code**											
							**1**	**2**	**3**	**4**	**5**	**6**	**7**	**8**	**9**	**10**	**11**	**12**
1	1.7	319.0453	319.0451	C_15_H_10_O_8_	0.90	Myricetin^a^	−	+	+	−	−	−	−	−	−	−	−	−
2	1.9	449.1083	449.1081	C_21_H_20_O_11_	0.57	Luteolin-7-glucoside	+	+	+	+	+	+	+	+	+	+	+	+
3	2.7	447.0927	447.0917	C_21_H_18_O_11_	1.60	Baicalin	−	−	−	−	−	+	+	+	+	+	−	−
4	3.0	431.1342	431.1342	C_22_H_22_O_9_	0.39	Ononin	+	−	−	−	−	−	−	−	−	−	−	−
5	5.9	611.1612	611.1611	C_27_H_30_O_16_	1.60	Rutin^a^	+	+	+	−	−	−	+	−	+	+	−	+
6	6.5	287.0555	287.0556	C_15_H_10_O_6_	1.21	Luteolin^a^	−	−	+	−	−	+	+	−	−	−	−	−
7	8.1	449.1083	449.1085	C_21_H_20_O_11_	1.23	Quercitrin^a^	+	+	+	+	+	−	−	+	−	−	+	−
8	8.12	171.0288	171.0288	C_15_H_10_O_5_	1.75	Gallic acid^a^	−	−	+	−	−	−	−	−	−	−	−	−
9	8.3	287.0555	287.0559	C_15_H_10_O_6_	1.50	Kaempferol^a^	−	+	−	−	−	−	−	−	−	−	−	−
10	7.5	301.0712	301.0713	C_16_H_12_O_6_	−1.78	Chrysoeriol	+	+	+	−	−	−	−	−	−	+	−	−
11	12.0	285.0763	285.0761	C_16_H_12_O_5_	−1.15	Ferulic acid	−	−	+		+	−		−	−	−	−	−
12	14.9	271.0606	271.0605	C_15_H_10_O_5_	0.10	Baicalein,	−	−	−	+	−	−	−	−	−	+	−	−

*RT, Retention Time; Cal. [M+H]^+^, calculated molecular weight of protonated molecule; Obs. [M+H]^+^, observed molecular weight of protonated molecule*;

a *Matched with standards; (+), Detected; (−), Not detected; Cal., Calculated; Obs, Observed; 1–15 are the plant extracts (1), A. augusta; (2), A. chinensis; (3), A. lebbeck; (4), B. ceiba; (5), C. arborea; (6), C. fragrans; (7), C. colebrookianum; (8), C. speciosus; (9), D. indica; (10), G. conyza; (11), H. sabdariffa; (12), M. charantia*.

The compounds Quercetin, Rutin, Kaempferol, Myricetin, Gallic Acid, and Luteolin were identified in the methanolic extract of the selected plants compared with retention time of authentic standards. Quercitrin was the dominant compounds in almost all methanolic extract, except in *C. fragrans, C. colebrookianum, D. indica*, and *G. conyza* with retention time 8.12 min (Table 3). Rutin was identified in eight plants out of 12 plants extract with retention time 5.90 min. Furthermore, Kaempferol was detected only in *A. chinensis* extract at retention time 8.3 min. On the other hand, other phenolic compounds have been detected in some plants- such as Myricetin found in *A. chinensis* and *A. lebbeck* plant at 1.7 min, Gallic acid detected only in *A. lebbeck* plant with 8.12 min and finally Luteolin was found in *A. lebbeck, C. fragrans* and *C. colebrookianum* with a retention time of 6.5 min (Table 3). Quercetin and rutin was detected in *A. augusta* and *H. Sabdariffa* plant.

Moreover, some compounds were unidentified due to lack of standards to determine the structure. Table 3 mentioned phenolic compounds with retention time, mass error (PPM), generated molecular formula using HPLC-DAD-ESI-TOF-MS were detected in the methanol extracts of the selected plants. One unidentified compounds C_21_H_20_O_11_ was detected in all plants extract except *C. colebrookianum*. In further studies, these unknown compounds identifications might prove to be important with the help of the reported unknown standards in this study.

### Antimicrobial activity of methanol extracts of selected medicinal plants

The results of antimicrobial assays exhibited that methanol crude extracts of 12 plants have greater antimicrobial activity against three human pathogenic bacteria (*S. aureus, P. aeruginosa*, and *E. coli*) and yeast (*C. albicans*) which are responsible for different food borne diseases. Three known antibiotics (ampicillin, streptomycin, and tetracycline) were used as positive control to compare with 12 medicinal plant extract. Our results showed that the plant extracts response were different against the bacteria tested. The methanol extract of dry leaves of 12 plants revealed the antibacterial activity against gram-positive (*S. aureus*) and gram-negative (*E. coli* and *P. aeruginosa*) bacteria at four different concentrations (1, 5, 7.5, and 10 mg/mL with DMSO). We found that out of 12 plants, seven plants showed positive activity against gram-positive bacteria with MIC values ranging from 1.635 to 7.972 mg/mL (Table 4). *A. lebbeck* and *M. charantia* had potent antibacterial activity against *S. aureus* with a MIC value of 7.972 and 7.634 mg/mL, respectively (Table 4). Five plants extracts (*A. augusta, A. chinensis, C. fragrans, C. speciosus*, and *H. sabdariffa*) showed no activity against *S. aureus*, while six plant extracts exhibited effect against gram negative bacteria (*E. coli* and *P. aeruginosa*) with the MIC values ranging from 5.621 to 7.815 mg/mL and 5.607 to 6.764 mg/mL respectively. *G. conyza* had highest activity against *P. aeruginosa* (6.764 mg/mL). Only three plant leaf extracts (*C. colebrookianum, D. indica*, and *M. charantia*) revealed positive antibacterial activity against *C. albicans* with the MIC values ranging from 6.293 to 6.896 (Table 4). However, *M. charantia* extract showed positive activity against all the pathogens tested.

**Table 4:** **Well diffusion assay of antimicrobial activity of the selected medicinal plant extracts**.

**Sample**	***Staphylococcus aureus***		***Pseudomonas aeruginosa***		***Candida albicans***		***Escherichia coli***	
	**Zone of inhibition (mm) using 10mg/ml extract**	**IC_50_ (mg/mL)**	**Zone of inhibition (mm) using 10mg/ml extract**	**IC_50_ (mg/mL)**	**Zone of inhibition (mm) using 10mg/ml extract**	**IC_50_ (mg/mL)**	**Zone of inhibition (mm) using 10mg/ml extract**	**IC_50_ (mg/ml)**
*A. augusta*	–	–	–	–	–	–	–	–
*A. chinensis*	–	–	–	–	–	–	–	–
*A. lebbeck*	6.00 ± 0.50^a^	7.972	–	–	–	–	7.5 ± 0.10^a^	5.621
*B. ceiba*	5.50 ± 0.25^bc^	1.635	5.0 ± 0.50^a^	6.728	–	–	8.5 ± 0.28^bc^	7.815
*C. arborea*	6.50 ± 0.28^bde^	4.939	8.00 ± 0.20^bc^	6.58	–	–	–	–
*C. fragrans*	–	–	8.00 ± 0.20^bc^	5.855	–	–	6.5 ± 0.20^bde^	6.111
*C. colebrookianum*	5.50 ± 0.25^bc^	4.139	–	–	7.00 ± 0.50^a^	6.293	9.0 ± 0.15^bdf^	6.634
*C. speciosus*	–	–	–	–	–	–	–	–
*D. indica*	6.00 ± 0.50^a^	5.139	4.0 ± 0.15^bde^	5.607	6.50 ± 0.28^bc^	6.896	–	–
*G. conyza*	5.00 ± 0.20^bdfg^	4.866	5.0 ± 0.50^a^	6.764	–	–	4.0 ± 0.28^bdfg^	6.647
*H. sabdariffa*	–	–	–	–	–	–	–	–
*M. charantia*	8.00 ± 0.20^bdfhi^	7.634	9.0 ± 0.28^bdfg^	6.759	9.00 ± 0.15^bde^	6.298	7.5 ± 0.10^a^	6.494
Ampicillin	10.50 ± 0.28^bdfhjk^	12.542	7.0 ± 0.20^bdfhi^	10.768	3.5 ± 0.10^bdfg^	9.612	14.50 ± 0.10^bdfhi^	14.821
Streptomycin	8.00 ± 0.50^bdfhi^	10.736	11.50 ± 0.28^bdfhjk^	14.321	2.0 ± 0.28^bdfhi^	8.704	11.50 ± 0.28^bdfhjk^	12.672
Tetracycline	10.00 ± 0.50^bdfhjk^	12.637	10.00 ± 0.15^bdfhjl^	12.735	2.5 ± 0.15^bdfhj^	8.431	12.00 ± 0.15^bdfhjl^	10.685
Fluconazole	–	–	–	–	6.5 ± 0.15^bc^	7.461	–	–

*Mean (±SD) followed by the same letter(s) in each column [Zone of inhibition (mm)] are not significantly different at P < 0.05 using Duncan's new multiple range test. – no activity shown*.

### Cytotoxicity screening by MTT assay against cell line

**Figure 2:**
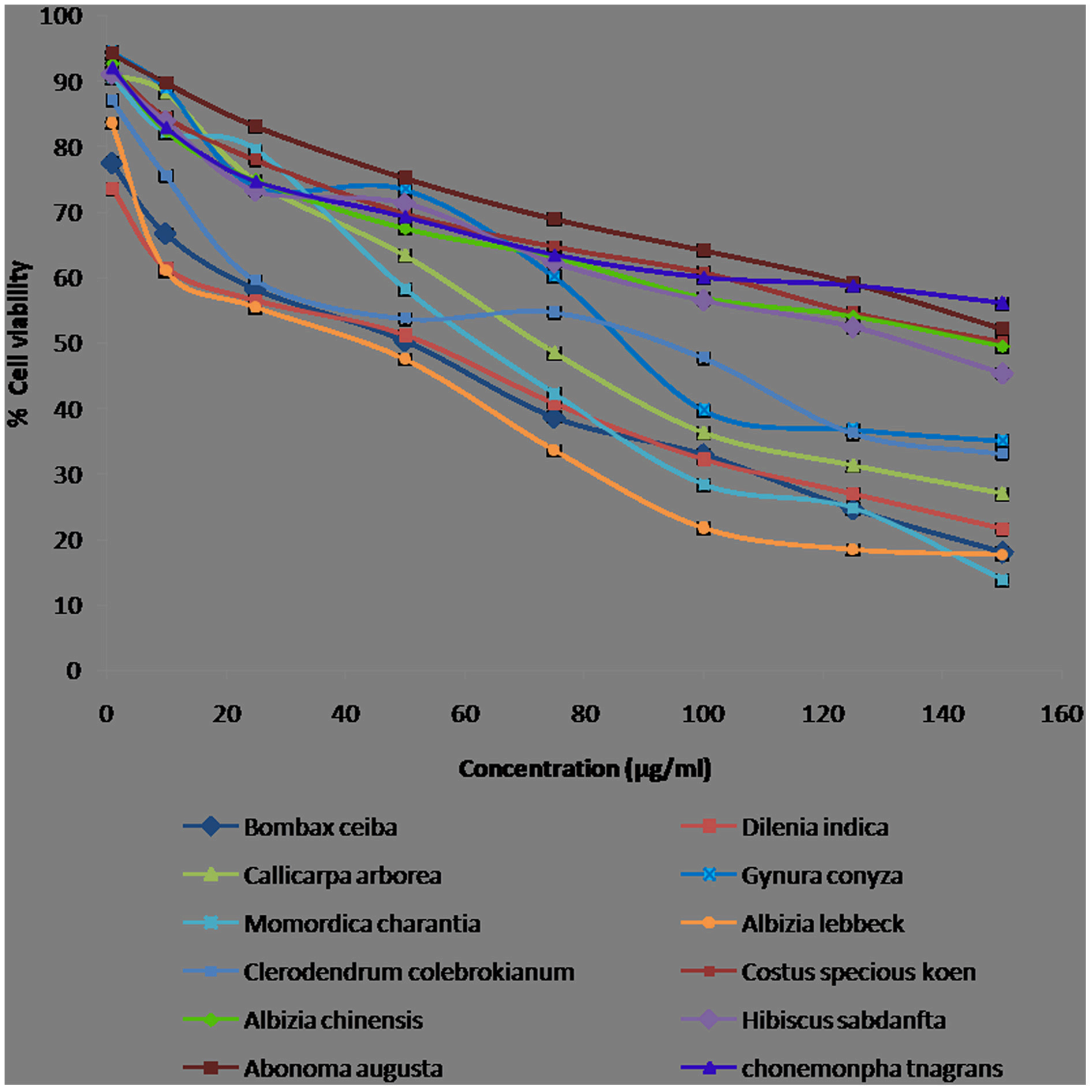
**Cell proliferation percent inhibition of methanolic leaf extracts from Mizoram on human hepatocarcinoma cell line (HepG2)**. Values are presented as mean ± SEM and are significant at *p* < 0.05.

The cytotoxic activity of the extract can be attributed to the different secondary metabolites present in its crude extract. In the present study, we have evaluated the cytotoxicity of crude methanolic leaf extracts of 12 medicinal plants at different concentrations (1, 10, 25, 50, 75, 100, 125, and 150 μg/mL) on HepG2 (human hepato carcinoma) cells using MTT [3-(4, 5-dimethylythiazol-2-yl)-2, 5-diphenyl-2H-tetrazolium hydrobromide] assay. The cytotoxic activities of the crude extracts were preliminarily screened by MTT assay, the percentage viability curves of treated cells were plotted against the extract concentrations, and the IC_50_ as compared to that of untreated cells was determined (Figure 2). All the extracts exhibited cytotoxic activity at various concentrations and some of the extracts (*A. lebbeck, D. indica*, and *B. ceiba*) had cytotoxicity at low concentrations with minor differences in IC_50_. Figure 2 shows the morphological variations such as shrinkage, nuclear condensation in the cells, which might be a probable indicator for apoptosis induced by the plant extract. Table 1 showed the cytotoxic activity (IC_50_) of the 12 plant's crude methanolic leaf extracts that are commonly used in the treatment of disease in Mizo traditional medicine. The ability of the extract to inhibit the proliferation of HepG2 cells at a low concentration makes it a possible potent chemotherapeutic agent. Among the plant extracts used for cytotoxic studies, the extracts of *A. lebbeck, D. indica*, and *B. ceiba* were found active on HepG2 cells (IC_50_ values 24.03, 25.09, and 29.66 μg/mL, respectively) and a significant decrease in cell viability was observed at low concentrations. *M. charantia, C. colebrokianum*, and *C. arborea* were found moderately active on HepG2 cells (IC_50_ values 56.77, 63.04, and 67.32 μg/mL, respectively) and *G. conyza* was at least active (IC_50_ values 86.55 μg/mL).

## Discussion

### Determination of total phenolics (TPC), flavonoids (TFC), and antioxidant activity by DPPH, ABTS, and reducing power assays

The maximum amount of TPC was recorded with the methanol extract of *B. ceiba* (181.91 μg of GAE/mg) followed by *D. indica* and *C. colebrookianum* with 180.15 and 158.37 μg of GAE/mg respectively. Jain et al. (2011) reported polyphenol content from aqueous extracts of *B. ceiba* as 30.95 μg GAE/mg. In this study, a significant TPC level has been obtained from a methanolic leaf extract of *B. ceiba* which is much higher than the reported earlier. Similar findings were recorded in the leaf extract of *D. indica* wherein the TPC content was found to be higher than the previously reported value of 25.25 mg GAE /g of plant extract (Saha et al., 2009). In the case of *C. colebrookianum*, a similar TPC value (104.4 mg GAE/100 mg) has been reported by Mandal et al. (2013). The highest TFC was found in *A. lebbeck* (102.2 μg of quercetin/mg). This was in accordance with the findings of Zia-Ul-Haq et al. (2013) who reported the TFC as 371.27 mg CAE/g and Malla et al. (2014) who found TFC as 22.48 mg quercetin/g. The TFC of *D. indica* was 98.25 μg of quercetin/mg which was again higher than the previously report by Islam et al. (2013) from the methanolic bark extract of *D. indica* (30.34 mg quercetin/g). To best our knowledge, this is first report of TFC from *B. ceiba* with significant flavonoid content of 88.84 μg quercetin/mg.

Apak et al. (2007) suggested that antioxidant activity cannot be estimated by using a single test. Hence, three parameters were used to strengthen our investigation. Namely, antioxidant activities of plants were examined as the free radical scavenging ability using DPPH, ABTS^+^, and reducing power. DPPH assay mainly depends on the hydrogen donating capacity to scavenge DPPH radicals. Free radicals play a major role in overcoming numerous chronic pathologies, such as cancer and cardiovascular diseases among others (Dorman et al., 2003). Antioxidants respond to the DPPH by reducing their number of DPPH molecules which in turn will be equivalent to the number of their OH^−^ ions prevailing. The reduced capacity of the DPPH radical as estimated at 515 nm indicates the decrease of absorbance by antioxidants action which will be proportional to the number of residual DPPH (Juan et al., 2005). The free radical scavenging property of DPPH can be noted as a change in color from purple to yellow when a DPPH electron binds to a radical scavenger forming reduced DPPH-H (Cai et al., 2003). DPPH result was expressed as IC_50_ (half maximal inhibitory concentration) value and the lower the value the better the antioxidant capacity.

In our study, we found that DPPH IC_50_ value of 29.96 μg/mL and ABTS IC_50_ value of 24.08 μg/mL in *D. indica* plant. These findings were comparable to the previously reported IC_50_ DPPH values of methanolic extracts of *D. indica* bark (12.32 μg /mL) by Alam et al. (2012). Similarly, *A. lebbeck* also showed lower IC_50_ DPPH value of 34.22 μg/mL and also observed ABTS IC_50_ value of 108.7 μg/mL. The IC_50_ DPPH value reported here in this study for *A. lebbeck* is extremely low compared to the previously reported values of 240 μg/mL of methanolic leaf extract (Malla et al., 2014). We found that IC_50_ DPPH value of 46.36 μg/mL and ABTS IC_50_ value of 89.19 μg/mL in *B. ceiba*. Similar reports by Jain et al. (2011) wherein an IC_50_ DPPH value of 15.07 μg/mL of extract was found from *B. ceiba* methanolic root extract. Nehete et al. (2010) showed IC_50_ DPPH value of 14.26 μg/mL for *C. speciosus* plant which was lower than our reported value.

Similarly, IC_50_ DPPH values for *A. augusta* (101.4 μg/mL) and *C. fragrans* (50 μg/mL) were reported by Hossain et al. (2015) and Shyma et al. (2013). This may be because this plant has more antioxidant compounds than other phytochemicals which is neutralizing the DPPH radical. As mentioned above, the best ABTS scavenging activity was shown in *D. indica* followed by *B. ceiba and A. lebbek*. The highest IC_50_ ABTS value was found in *C. speciosus* (513.4 μg/mL of extract) which was in deviation to that of previous reports by Vijayalakshmi and Sarada (2008), who mentioned that IC_50_ ABTS value of 85.51 μg/mL of methanolic leaf extract of *C. speciosus* plant. Thus, by all three assays used five plants (*D. indica, A. lebbeck, B. ceiba, C. arborea*, and *C. colebrookianum*) have shown significant antioxidant capacity. Correlation of TFC with respect to reducing power (OD) values indicated an *R*^2^ of 0.541; which is in accordance to the correlation value of TPC to RPA (reducing power assay). Thus, these positive correlations between the TPC content which inturn is dominated by TFC to that of the reducing and antioxidant assays indicates that the flavonoids and polyphenols may be responsible for the antioxidant properties (Zhao et al., 2006).

### Detection of phenolic compounds by HPLC-DAD-ESI-TOF-MS

To the best of our knowledge, this is the first report about phenolic compounds detected by HPLC analysis for plant species like *A. chinensis, A. lebbeck, B. ceiba, C. arborea, C. fragrans, C. colebrookianum, C. speciosus, Gynura conyza*, and *M. charantia*. Previous findings reported the presence of phenolic compounds in *A. augusta* and *H. sabdariffa* leaves (Borrás-Linares et al., 2015; Khanra et al., 2015; Zhen et al., 2016). Quercetin and Rutin was detected in *A. augusta* and *H. sabdariffa* which was similarly reported in Borrás-Linares et al. (2015) and Khanra et al. (2015). However, Luteolin, a phenolic compound, was identified for the first time in *C. colebrokianum* plant. On the other hand, *H. sabdariffa* plant was found to possess only Quercetin and Rutin compounds which were reported earlier by Borrás-Linares et al. (2015).

### Antimicrobial activity of methanol extracts of selected medicinal plants

Antimicrobial assays of methanolic extract of 12 plants showed strong activity against three human pathogenic bacteria (*S. aureus, P. aeruginosa*, and *E. coli*) and yeast *C. albicans* which are responsible for different food borne diseases. Six plant extract indicated positive activity against *E. coli* and *P. aeruginosa* with MIC values ranging from 5.621 to 7.815 mg/mL and 5.607 to 6.764 mg/mL, respectively. This finding is similarly reported by Malla et al. (2014); Mahomoodally et al. (2010); Mandal et al. (2015). *B. ceiba* showed highest antibacterial activity against *E. coli* (7.815 mg/mL). Similar results were reported by Digge et al. (2015). From our results, different ranges of MIC values were found in all plants tested as reported by Yaldiz et al. (2015), but to the best of our knowledge, this is the first time report of three plants viz. *C. colebrookianum* and *G. conyza* with antimicrobial activity against *S. aureus, E. coli, P. aeruginosa*, and *C. albicans*. All the plants which showed positive antimicrobial activity can be used to characterize and develop new medicinal compounds or pharmaceutical drug to control human pathogenic bacterial disease (Higginbotham et al., 2014; Borrás-Linares et al., 2015).

Plant polyphenols are considered to have antimicrobial activity, generally by the disturbance of the function of bacterial cell membranes which retards bacterial growth or multiplication. Nevertheless, other compounds such as Quercetin, could act essentially by enzyme inhibition of DNA gyrase (Cushnie and Lamb, 2005). However, according to the antimicrobial activity of plant extracts, a higher total content in phenolic compounds including flavonoid, cyanidin, and delphinidin are not always correlated to high antibacterial activity. In fact, the most potent plant extracts against the studied microorganisms did not exhibit the highest content in these compounds, such as *A. lebbeck, B. ceiba, G. conyza*, and *M. charantia*. Therefore, the antibacterial activity exhibited by these extracts could be attributed to the presence of specific phenolic compounds in their composition and to the possible existence of synergistic effects with other non phenolic compounds present in the extracts.

### Cytotoxicity screening by MTT assay against cancer cell line

Plant extracts contain almost unlimited compounds and have the capacity to produce cytotoxicity that fascinates researchers in the quest for new and novel therapeutic drugs (Jain and Jain, 2011). The persistency search for new compounds in medicinal plant and traditional food is a realistic and promising strategy for prevention of diseases (Li et al., 2012). We found that the cytotoxicity test of 12 methanolic plant extracts at different concentrations (1, 10, 25, 50, 75, 100, 125, and 150 μg/mL) on HepG2 (human hepato carcinoma) cells using MTT assay. According to the United States National Cancer Institute plant screening program, a crude extract is generally considered to have *in vitro* cytotoxic activity if the IC50 is <30–40 μg/mL (Oskoueian et al., 2011). Three plant extracts (*A. lebbeck, D. indica*, and *B. ceiba*) showed cytotoxicity activity at very low concentrations. Similarly, the potent cytotoxicity of crude methanolic leaf extract of *A. lebbeck* against breast cancer cell line (MCF-7) and a slight inhibition of HT-29 cell line were reported (Aditya et al., 2014).

## Conclusions

The results of the study showed that the leaves of the selected traditional medicinal plants have antioxidant and antimicrobial activities. The results highlighted the potential of the leaves of the selected plants as source of natural antioxidant and antimicrobials. Furthermore, this work is the first report available for the cytotoxicity assay against HepG2 (human hepato carcinoma) cells and phenolic compounds from the selected plants. This report can lead to explore the potential of medicinal plants from Northeast India as a valuable source for drug discovery.

## Author contributions

GS, Complete the entire experiments and prepared the draft manuscript. AP, VL, Supported GS to fulfill the experiment and also help in preparing the manuscript. VM, SS, Performed the cytotoxicity assay. BS, NK, VG, All the experiment checked carefully, written the manuscript and approved the final manuscript. BK, SK, HL, performed HPLC analysis.

### Conflict of interest statement

The authors declare that the research was conducted in the absence of any commercial or financial relationships that could be construed as a potential conflict of interest.
